# Evaluation of right atrial function using right atrial speckle tracking analysis in patients with pulmonary artery hypertension

**DOI:** 10.1007/s12574-015-0270-4

**Published:** 2015-11-27

**Authors:** Konomi Sakata, Yoichiro Uesugi, Aoi Isaka, Toshinori Minamishima, Kenichi Matsushita, Toru Satoh, Hideaki Yoshino

**Affiliations:** Division of Cardiology, Second Department of Internal Medicine, Kyorin University School of Medicine, 6-20-2 Shinkawa, Mitaka, Tokyo 181-8611 Japan

**Keywords:** Speckle tracking imaging, Pulmonary artery hypertension, Right atrial strain, Right atrial function

## Abstract

**Background:**

In patients with pulmonary artery hypertension (PAH), right ventricular pressure overload eventually causes right heart failure (RHF), leading to a poor prognosis. Right atrial (RA) overload and RA dysfunction occur in patients with PAH-complicated RHF.

**Objectives:**

We evaluated RA function using right atrial longitudinal strain (RALS) by two-dimensional speckle tracking echocardiography (2D-STE) and investigated the association between RALS and the severity of RHF in patients with pulmonary artery hypertension (PAH) noninvasively.

**Methods:**

We performed 2D-STE in 56 PAH patients and 20 normal control subjects. The peak global RALS and peak global RA longitudinal strain rate (RALSR) were analyzed by 2D-STE. Simultaneous right heart catheterization was performed to determine the right atrial pressure (RAP) and cardiac index (CI).

**Results:**

Peak global RALS (34.6 ± 14.1 vs. 58.3 ± 9.9 %, *p* < 0.0001) and peak global RALSR (2.5 ± 1.3 vs. 3.1 ± 1.2 s^−1^, *p* < 0.0001) were significantly lower in PAH patients compared with normal controls. There was a significant negative correlation between peak global RALS and RAP (*r* = −0.8037, *p* < 0.0001). There was a significant positive correlation between peak global RALS and CI (*r* = 0.8179, *p* < 0.0001). Peak global RALSR was also correlated with RAP (*r* = −0.7308, *p* < 0.0001) and CI (*r* = 0.7596, *p* < 0.0001).

**Conclusions:**

RALS and RALSR by 2D-STE were useful for noninvasive evaluation of RA dysfunction and the severity of RHF in patients with PAH.

## Introduction

In patients with pulmonary artery hypertension (PAH), the pulmonary vascular resistance (PVR), pulmonary artery pressure (PAP), and right ventricular (RV) afterload are all increased. The increase of RV afterload causes RV dilatation and hypertrophy, and impaired compliance of the hypertrophied and dilated RV contributes to a further increase of RV end-diastolic pressure and right atrial pressure (RAP), eventually leading to right heart failure (RHF) and a poor prognosis. In fact, RHF is the main cause of death in patients with PAH. RAP and the cardiac index (CI) are parameters with established usefulness for assessing the severity of RHF and prognosis of PAH patients [1]. Assessment of RA overload and RA dysfunction has important implications for evaluating the severity of RHF in the management of patients with PAH. Right heart catheterization is usually performed to measure RAP and evaluate the severity of PAH. Among noninvasive methods, RAP is commonly estimated from two-dimensional echocardiographic (2DE) parameters, such as the presence of inferior vena cava (IVC) dilation and extent of inspiratory IVC collapse [2–4], but the reliability of these parameters has varied in previous studies. IVC-based estimates of RAP are less reliable than invasive values. In addition, the estimation of IVC is not always easily obtained because of the anatomical differences of each patient [2–6]. A previous study indicated that the addition of the three-dimensional maximal right atrial volume index (RAVI) to IVC parameters may be helpful for noninvasive estimation of RAP in patients with heart failure due to left ventricular systolic dysfunction [7]. However, that study focused on the relation of RA size to RAP and did not include assessment of RA function or the relation with the CI. The important influence of the progression of RHF on the outcome of PAH has been confirmed by the known prognostic impact of the RAP and CI. Noninvasive and quantitative 2DE parameters estimating invasive hemodynamic parameters (including RAP, CI, and PVR) could be useful to evaluate the severity of RHF in patients with PAH and to monitor their response to therapy.

Two-dimensional speckle tracking echocardiography (2D-STE) is a reliable technique for angle-independent tracking of myocardial deformation that allows noninvasive and quantitative assessment of global or regional myocardial function [8, 9]. This method is potentially able to explore RA deformation during each phase of the cardiac cycle. A previous study showed that the peak right atrial longitudinal strain (RALS) measured by 2D-STE at the end of the RA reservoir phase is correlated with the invasively measured systolic PAP and that assessment of RALS by 2D-STE could predict pulmonary hypertension in patients with heart failure due to left ventricular systolic dysfunction [10]. Another study showed that analysis of left atrial longitudinal deformation by 2D-STE was well correlated with the pulmonary capillary wedge pressure (PCWP), providing a better estimate of left ventricular filling pressure in patients with left ventricular dysfunction [11]. Similarly, measuring RALS by 2D-STE may be useful for the estimation of RAP and evaluation of RA function in patients with PAH. Although 2D-STE is widely used, there have been few reports about the assessment of RA overload and RA dysfunction in PAH patients without left heart disease based on measurement of RALS by 2D-STE. The purpose of the present study was to investigate RA dysfunction in PAH patients without left heart disease by measuring RALS with 2D-STE as a noninvasive and quantitative method of assessment.

## Methods

### Study population

We investigated 56 patients with PAH (mean age 47.4 ± 15.6 years, 46 females) and 20 healthy control subjects (mean age 38 ± 12 years, 12 females). According to current recommendations, PAH was defined as a resting mean pulmonary artery pressure >25 mmHg and with a normal pulmonary capillary wedge pressure (≤15 mmHg) [12, 13]. Patients who were not in sinus rhythm, those with a prosthetic tricuspid valve or annuloplasty, and those with left heart disease were excluded from this study. All patients provided written informed consent to participation in the study, and its protocol was approved by the institutional review board and ethics committee of our hospital.

### Echocardiography

Echocardiography was performed with an Artida ultrasound system (Toshiba Medical Systems, Tochigi, Japan). Transthoracic two-dimensional echocardiography was done using a 3-MHz/PST-30SBT transducer. Images of the RV and RA were obtained in the apical four-chamber view during breath-holding and with a stable ECG recording. These images were obtained while taking care to capture the entire RA, allowing for more reliable delineation of the atrial endocardial border. The frame rate was set at between 60 and 80/s. Three cardiac cycles recorded at each plane were stored in cine loop format in order to subsequently select the images with the best quality for off-line speckle tracking analysis. The same transducer (1.7–3.6 MHz) was used with both systems, and special care was taken to employ a similar sector width, focus position, and frame rate range during image acquisition. The RV end-diastolic area index (RVEDAI: RVEDA/body surface area) and RV end-systolic area index (RVESAI: RVESA/body surface area) were measured in the apical four-chamber view to calculate the RV fractional area change [(%RVFAC) = (RVEDA – RVESA)/RVEDA × 100 %] [14, 15]. The two-dimensional maximal RA volume was calculated by Simpson’s method in the apical four-chamber view at end-systole and was adjusted for body surface area to obtain the 2D maximal RA volume index (RAVI) [16, 17].

The IVC diameter was measured on 2DE images at a site approximately 2 cm from its junction with the right atrium. Maximal and minimal IVC diameters during the respiratory cycle (with a sniff test) were measured to calculate the extent of IVC collapse during respiration.

### Two-dimensional speckle tracking analysis

**Fig. 1:**
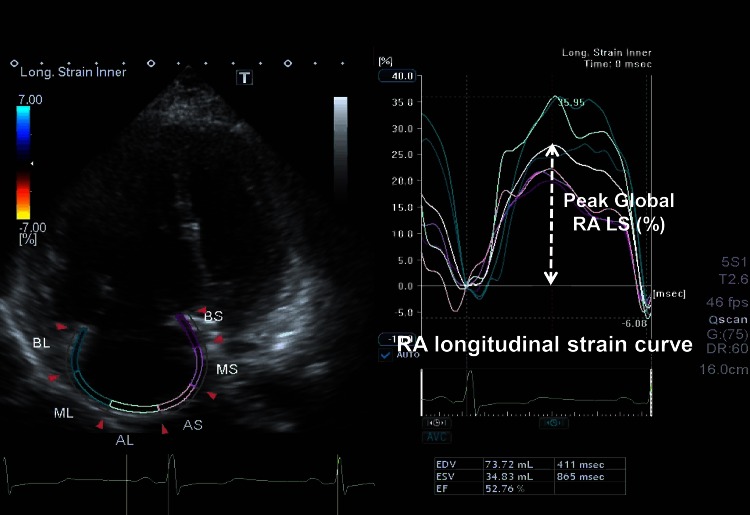
Right atrial longitudinal strain and strain rate determined by 2D speckle tracking echocardiography (2D-STE). The right atrial longitudinal strain and strain rate determined by 2D speckle tracking echocardiography (2D-STE): the right atrial (RA) wall was divided into six segments automatically. Peak right atrial longitudinal strain (RALS) and systolic strain rate (RALSR) were measured in each segment at the mid-RA free wall and interatrial septum, and global values were calculated

Speckle tracking analysis was performed by a single experienced and independent investigator, who was blinded to the invasive hemodynamic data, using commercially available semiautomated 2D-STE software for a Toshiba system (2D Wall Motion Tracking, Toshiba Medical Systems). The RA endocardial border was manually traced in the four-chamber view, followed by manual tracing of the epicardial border, thus delineating a region of interest composed of six segments. After analysis of segmental tracking quality and manual adjustment of the region of interest, longitudinal strain curves were generated for each atrial segment by the software. RA longitudinal strain (RALS) was measured in six segments of the RA. As previously described, peak RALS at the end of the RA reservoir phase was calculated by averaging the values for all RA segments to yield the global RALS. The RA longitudinal strain rate (RALSR) was also measured in each of the six segments, and the average for all six segments was calculated (global RALSR) (Fig. 1) [8, 10, 11, 18].

### Cardiac catheterization

Invasive measurement of cardiac pressures was performed by an investigator who was blinded to the echocardiographic data. The median time difference between echocardiography and right heart catheterization was 4 h (interquartile range 2–5 h). The pressure transducers were balanced before data acquisition with the zero level being set at the mid-axillary line. Right heart catheterization was done to measure the mean PAP, mean RAP, and mean PCWP. The position of the wedge was verified by fluoroscopy, phasic changes of pressure waveforms, and measurement of oxygen saturation. Cardiac output (CO) and the cardiac index (CI) were derived by the direct Fick method as the average of five cardiac cycles with <10 % variation. PVR was calculated by the following formula: PVR = (mean PAP – PCWP)/CO.

The serum BNP concentration was also measured. Natural log (Ln) transformation of BNP values was performed because of their skewed distribution.

### Statistical analysis

Continuous variables are expressed as the mean ± SD, and categorical variables are expressed as percentages. Student’s *t* test and the chi-square test were used for comparison of data as appropriate. The relations between echocardiographic parameters acquired by speckle tracking analysis and invasive data were assessed by calculation of Pearson correlation coefficients. A two-tailed *p* value <0.05 was considered to indicate statistical significance in all analyses. Intraclass correlation coefficients were calculated to examine intra- and interobserver variability. Analysis was performed with SPSS software for Macintosh (version 17; SPSS, Inc., Chicago, IL, USA).

## Results

### Patient characteristics

The major clinical and hemodynamic parameters for the study population are listed in Table 1. Fifty-six patients with PAH (46 females and 10 males with a mean age of 47.4 ± 15.6 years) were enrolled in the study. The etiology of PAH was connective tissue disease in 13, HIV infection in 1, and idiopathic pulmonary arterial hypertension in 42 patients. Thirteen patients were in NYHA classes III or IV (23 %: NYHA III in 9 patients, NYHA IV in 4 patients) (Table 1).

**Table 1:** Baseline characteristics and catheterization data

	Mean ± SD [range]
Age (years)	47.4 ± 15.6 [22–67]
Sex, male/female	10/46
Idiopathic PAH	42 (75 %)
PAH due to connective tissue disease	13 (23 %)
HIV infection	1 (2 %)
NYHA III/IV	9 (16 %)/4 (7 %)
Heart rate (bpm)	79.4 ± 17.9
Serum BNP (pg/ml) [median (interquartile range)]	79 [30–250]
Right heart catheterization data	
Systolic PAP (mmHg)	72.2 ± 19.6 [33–115]
Mean PAP (mmHg)	43.0 ± 11.5 [25–76]
Mean RAP (mmHg)	7.8 ± 6.0 [2–30]
PCWP (mmHg)	8.6 ± 3.6 [3–15]
PVR (mmHg/l/min)	10.0 ± 6.2 [1–33]
CO (l/min)	4.2 ± 1.9 [1.4–11.6]
CI (l/min/m^2^)	2.8 ± 1.2 [1.0–6.3]
Medications	
Endothelin receptor antagonists	40 (71 %)
Phosphodiesterase 5 inhibitors	46 (92 %)
Epoprostenol	27 (48 %)
Diuretics	30 (54 %)

Values are the mean ± SD [range] or number

*HR* heart rate, *BNP* brain natriuretic peptide, *PAP* pulmonary artery pressure, *PCWP* pulmonary capillary wedge pressure, *PVR* pulmonary vascular resistance, *RAP* right atrial pressure, *CO* cardiac output, *CI* cardiac index

Right heart catheterization showed that the systolic PAP was 72.2 ± 19.6 mmHg (range 33–115 mmHg), mean PAP (MPAP) was 43.0 ± 11.5 mmHg (range 25–76 mmHg), mean RAP was 7.8 ± 6.0 mmHg (range 2–30 mmHg), CI was 2.8 ± 1.2 l/min/m^2^ (range 1.0–6.3 l/min/m^2^), and PCWP was 8.6 ± 3.6 mmHg. Serum BNP was median 79 pg/ml (interquartile range 30–250 pg/ml). LVEF was 70.2 ± 6.6 %, and all patients had normal left ventricular function.

Of the 56 patients, 27 (48 %) were on a prostacyclin analog at the time of echocardiography. There were 40 patients (71 %) on an endothelin receptor antagonist, 46 patients (92 %) on a phosphodiesterase-5 inhibitor, and 30 patients (54 %) on a diuretic. We offered optimal combinations of targeted therapies.

### RV and RA dimensions and function by two-dimensional echocardiography

RV and RA dimensions and function were evaluated by 2DE (Table 2). The right ventricular end-diastolic area index (RVEDAI) (18.6 ± 5.5 vs. 10.9 ± 1.9 cm^2^/m^2^, *p* < 0.0001) and right ventricular end-systolic area index (RVESAI) (12.3 ± 5.9 vs. 5.8 ± 1 cm^2^/m^2^, *p* < 0.0001) were significantly larger in the PAH patients compared with the normal controls, while the right ventricular fractional area change (%RVFAC) (34.4 ± 10.5 vs. 50.5 ± 6.5 %, *p* < 0.0001) was significantly smaller. Evaluation of RA size and function by 2DE revealed that maximal RAVI (38.1 ± 26.5 vs. 14.5 ± 2.1 ml/m^2^, *p* < 0.0001) was significantly larger in the PAH patients compared with the normal controls. Maximal RAVI showed significant correlations with RAP (*r* = 0.6789, *p* < 0.0001) and BNP (*r* = 0.5646, *p* < 0.0001), but not with CI (*r* = −0.2944, *p* = 0.05), PVR (*r* = 0.2652, *p* = 0.1711), and systolic PAP (*r* = 0.2278, *p* = 0.1860).

**Table 2:** Echocardiographic parameters

	PAH patients	Normal control subjects	*p* value
Two-dimensional echocardiography			
LVEF (%)	70.2 ± 6.6	67.4 ± 6.0	0.1061
RVEDAI (cm^2^/m^2^)	18.6 ± 5.5	10.9 ± 1.9	<0.0001
RVESAI (cm^2^/m^2^)	12.3 ± 5.9	5.8 ± 1.0	<0.0001
%RVFAC (%)	32.0 ± 11.5	50.5 ± 6.5	<0.0001
Maximal RAVI (ml/m^2^)	38.1 ± 26.5	14.5 ± 2.1	<0.0001
IVC (cm)	15.8 ± 6.0	10.2 ± 4.0	0.0056
Moderate or severe TR by color Doppler	24 (43 %)	0 (0 %)	<0.0001
TRPG (mmHg)	77.3 ± 24.9	21.7 ± 4.0	<0.0001
Estimated PASP (mmHg)	80.5 ± 26.0	30.6 ± 3.0	<0.0001
RA speckle tracking analysis			
Peak global RALS (%)	34.6 ± 14.1	58.3 ± 9.9	<0.0001
Peak global RALSR (s^−1^)	2.5 ± 1.3	3.5 ± 2.0	<0.0001

*LVEF* left ventricular ejection fraction, *RVEDAI* right ventricular end-diastolic area index, *RVESAI* right ventricular end-systolic area index, *%RVFAC* RV fractional area change, *RAEDVI* right atrial end-diastolic volume index, *RAESVI* right atrial end-systolic area index, *RAEF* right atrial ejection fraction, *IVC* inferior vena cava, *TR* tricuspid regurgitation, *TRPG* pressure gradient of tricuspid regurgitation measured by continuous-wave Doppler echocardiography, *TAPSE* tricuspid annular plane systolic excursion, *RALS* right atrial longitudinal strain, *IAS* interatrial septum, *RALSR* right atrial longitudinal systolic strain rate

### RA strain and strain rate by 2D-STE

Peak global RALS (34.6 ± 14.1 vs. 58.3 ± 9.9 %, *p* < 0.0001) was significantly lower in the PAH patients compared with the normal control subjects. There were also significant differences of peak global RALSR (2.5 ± 1.3 vs. 3.5 ± 2.0 s^−1^, *p* < 0.0001) between the PAH patients and normal controls (Table 2).

Analysis of RALS intraobserver variability demonstrated an intraclass correlation coefficient of 0.94, and analysis of interobserver variability yielded an intraclass correlation coefficient of 0.89.

### Correlations between 2D-STE and hemodynamic parameters and 2D-echocardiographic parameters

**Fig. 2:**
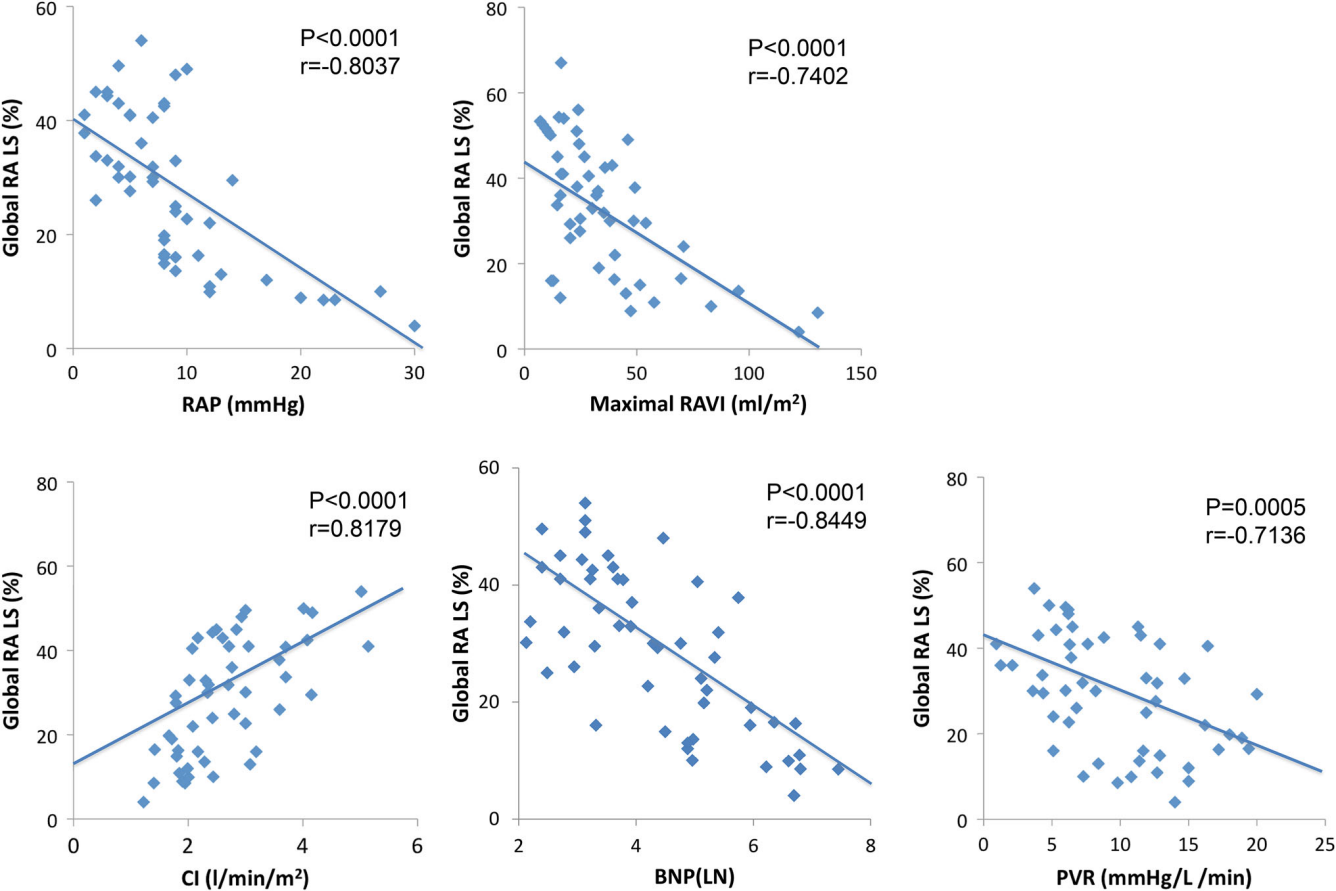
Correlation between the RALS and hemodynamic parameters. Correlations between the global peak right atrial longitudinal strain (RALS) and right atrial pressure (RAP), maximal right atrial volume index (RAVI), cardiac index (CI), brain natriuretic peptide (BNP), and pulmonary vascular resistance (PVR)

Peak global RALS showed a negative correlation with RAP (*r* = −0.8037, *p* < 0.0001), a positive correlation with CI (*r* = 0.8179, *p* < 0.0001), a negative correlation with PVR (*r* = −0.7136, *p* = 0.0005), and a negative correlation with BNP (*r* = −0.8449, *p* < 0.0001). There was a significant correlation between peak global RALS and maximal RAVI (*r* = −0.7402, *p* < 0.0001) (Fig. 2). The correlation between peak global RALS and systolic PAP was very weak (*r* = −0.3052, *p* = 0.0498).

There were significant correlations between peak global RALS and RVEDAI (*r* = 0.5457, *p* < 0.0001), %RVFAC (*r* = 0.5158, *p* = 0.0003), and TAPSE (*r* = 0.5665, *p* < 0.0001). There was a significant difference in peak global RALS between mild TR patients and moderate or severe TR patients (31.7 ± 12.8 vs. 25.2 ± 13.5 %, *p* = 0.0405). There was no significant correlation between TR severity and peak global RALS.

**Fig. 3:**
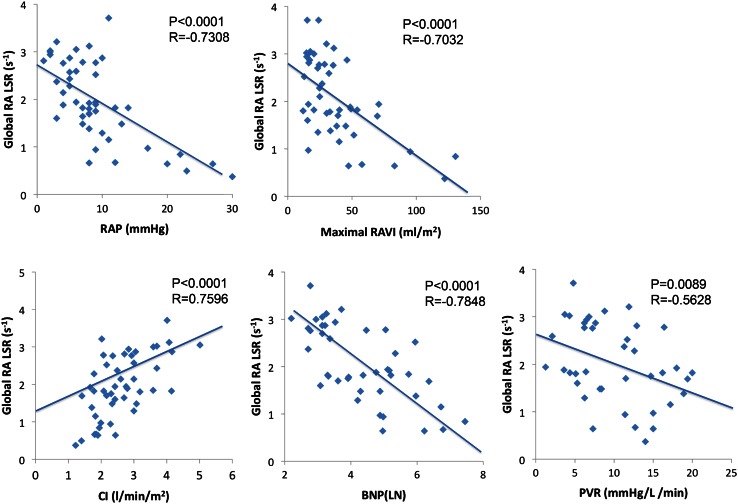
Correlation between the RALSR and hemodynamic parameters. Correlations between the global peak right atrial longitudinal strain rate (RALSR) and right atrial pressure (RAP), maximal right atrial volume index (RAVI), cardiac index (CI), brain natriuretic peptide (BNP), and pulmonary vascular resistance (PVR)

Peak global RALSR showed a significant positive correlation with CI (*r* = 0.7596, *p* < 0.0001) and significant negative correlations with RAP (*r* = −0.7308, *p* < 0.0001), PVR (*r* = −0.5628, *p* = 0.0089), and BNP (*r* = −0.7848, *p* < 0.0001). Peak global RALSR also showed a significant correlation with maximal RAVI (*r* = −0.7032, *p* < 0.0001) (Fig. 3). There was no significant correlation of systolic PAP with peak global RALSR.

## Discussion

### Echocardiographic versus right heart catheterization findings

This was the first study to examine the utility of 2D-STE for noninvasive evaluation of RA overload, RA dysfunction, and the severity of RHF in PAH patients. Our findings showed a good correlation between the RA parameters measured by 2D-STE and invasive hemodynamic data.

PAH is a progressive and debilitating disease caused by persistent RV pressure overload, such as that due to an increased PVR and pulmonary artery pressure (PAP). In patients with PAH, RV remodeling (dilatation and hypertrophy) is a complex set of functional and structural adaptations that occur in response to chronic pressure overload. The ability of the right ventricle to sustain its stroke volume and cardiac output despite an increased pressure load determines the severity of a patient’s symptoms and is also one of the most important factors impacting the survival of patients with PAH [19–21]. In this study, 23 % of the PAH patients investigated had severe RHF (NYHA classes III or IV), and right heart catheterization revealed an increase of PVR, PAP, and RAP, as well as a decrease of the CI. Standard 2D echocardiography showed RA enlargement, which was characterized by an increase of the maximal RAVI. There was also an increase of RVEDAI and RVESAI, as well as a decrease of %RVFAC, indicating the presence of RV dilatation and RV dysfunction. Because our subjects included patients with PAH complicated by severe RHF, their RV function decreased along with the increase of RV afterload caused by elevation of PVR and RV dilatation, leading to an increase of RAP and maximal RAVI that indicated RA overload. As RAP and maximal RAVI both increased, RALS and RALSR (the parameters of myocardial function measured by 2D-STE) both decreased. The present study thus demonstrated that the extent of RA overload and RA dysfunction could be evaluated by 2D-STE in PAH patients.

### Importance of RA function in PAH

RV systolic function is initially preserved in the face of RV pressure overload, but diastolic dysfunction occurs as a consequence of myocardial hypertrophy and dilatation. With time, the ventricle also thickens and its filling becomes more dependent on RA performance, which means that RHF can occur if atrial function is sufficiently impaired. It is likely that RA dysfunction contributes heavily to the occurrence of RHF in PAH patients.

As RV dysfunction progresses because of RV pressure overload, the preload reserve responds by the Frank-Starling mechanism, and the atrium compensates for ventricular dysfunction to maintain cardiac output by augmentation of RA ejection through the increase of both RA pressure and RA volume. However, when RV pressure overload progresses further and the preload reserve reaches its limit, atrial compensation for the increased RV afterload is lost, leading to a decrease of cardiac output with the onset of severe RHF and rapid deterioration until death occurs [22, 23]. Due to the significant contribution of RA function to the preload reserve, it is considered that as the RV pressure overload worsens in PAH patients, the role of the right atrium grows more important, so that its influence on RHF and the prognosis becomes greater. In other words, when RAP and RA dimensions have increased to their effective limits, RA compensation for RV dysfunction will be impaired, leading to a decrease of cardiac output and aggravation of RHF. In the present study, peak global RALS and peak global RALSR measured by 2D-STE showed significant negative correlations with PVR, and both parameters decreased as the PVR and RV afterload showed an increase. In addition, the RAP and maximal RAVI both displayed significant negative correlations with peak global RALS and peak global RALSR, which decreased as the preload reserve increased. Furthermore, peak global RALS and peak global RALSR both had a strong positive correlation with CI, indicating that a decrease of CI might be associated with impairment of RA compensation for RV dysfunction. Thus, peak global RALS and peak global RALSR are noninvasive parameters for quantitative evaluation of RA function, including RA overload and RA compensation for RV dysfunction, which can be used as indexes for assessing the severity of RHF in PAH patients.

### Evaluation of RA function with 2D-STE

Measurement of RA dimensions by 2D and 3D echocardiography is used for the evaluation of RA function [24, 25]. It has been reported that the RA volume index measured by 3D echocardiography in conjunction with inferior vena cava parameters has a high accuracy for detecting the elevation of RAP in patients with left heart failure [7]. In the present study, not only RA dimensions but also 2D-STE was utilized to evaluate both RA function and RAP.

Speckle tracking imaging (STI) is a reliable method for angle-independent and objective quantification of myocardial deformation during each phase of the cardiac cycle using 2DE. Strain is defined as the percent change of myocardial deformation, while its derivative (the strain rate) represents the rate of deformation of the myocardium over time. The strain rate has been shown to be closely correlated with myocardial contractility by both in vitro and in vivo experimental studies [26].

The right atrium has multiple roles during the cardiac cycle, since it (1) provides a reservoir for systemic venous return when the tricuspid valve is closed, (2) provides a passive conduit for bleed during early diastole when the tricuspid valve opens, and (3) actively expels blood during late diastole with atrial contraction [25]. Gaynon et al. demonstrated experimentally that the right atrium can act more or less as a reservoir than a conduit with dynamic adjustment of its role [27]. In a PAH model with partial pulmonary artery occlusion, the early RA conduit function decreased and RA reservoir function showed a slight increase, resulting in a decrease of the early conduit-to-reservoir ratio. Cardiac output was inversely related to the conduit-to-reservoir ratio, and the relative contribution of RA reservoir function increased along with the increase of cardiac output. The RA reservoir phase is a dynamic rather than static phase of the cardiac cycle, and RA deformation is dependent on PVR and PAP, which act on the right ventricle and consequently influence the atrium. Therefore, we evaluated RA function in our patients with PAH by focusing on the reservoir phase. 2D-STE evaluation of RA function is feasible throughout the cardiac cycle, and peak RALS identifies the reservoir phase. Padeletti et al. reported that RALS was the strongest predictor of pulmonary hypertension in patients with heart failure and left ventricular dysfunction and was inversely correlated with PAP and PVR, suggesting that RA function declines with an increase of pulmonary pressure and vascular resistance [10]. The present study investigated patients who had PAH without left ventricular disease and mainly focused on the reservoir phase of RA function assessed by 2D-STE, allowing the evaluation of RA overload and RA dysfunction.

We demonstrated that peak global RALS and peak global RALSR showed significant inverse correlations with RAP and maximal RAVI, as well as PVR. In previous reports, peak global RALS and peak global RALSR were also inversely correlated with PVR. In addition, the peak global RALS and peak global RALSR both decreased progressively with the increment of RAP and RA dilatation. Moreover, peak global RALS and peak global RALSR were closely correlated with the CI. Therefore, peak global RALS and peak global RALSR can be used as parameters for the evaluation of RA overload and RA dysfunction in PAH. Peak global RALS may even be a factor affecting the prognosis of PAH since it showed strong correlations with all of RAP, CI, and BNP, which are known prognostic indicators for pulmonary hypertension.

The present study demonstrates little correlation between PAP and peak global RALS. It was previously reported that RALS showed a significant correlation with systolic PAP in patients who had chronic systolic heart failure complicated by left ventricular dysfunction [10]. This difference may have arisen because that study included patients with PAH complicated by left ventricular systolic dysfunction, but did not include PAH patients with severe RHF. In some patients with progressive RHF, PAP may actually decrease as a consequence of low cardiac output [28]. The above-mentioned study included patients with a mean systolic PAP of 43 ± 16 mmHg, which was lower than that of the PAH patients in our study (mean systolic of 73 ± 19 mmHg) and also did not include patients complicated by severe RHF. Whereas evaluation of the severity of PAH based on PAP might be difficult for this reason, peak global RALS and peak global RALSR measured noninvasively by 2D-STE can be utilized to quantitatively evaluate right heart function in PAH patients with severe RHF.

### Limitations

Padeletti et al. [10] divided the RA wall into six segments to measure RALS and reported that it increased in the order of basal, mid, and apical segments and that septal values were higher than lateral values, so that peak RALS varies depending upon where it is measured. Our study showed similar results using global peak RALS, which we calculated as the average of the peak RALS values for six RA segments to provide an index for the function of the entire RA since the peak RALS varies among segments. Further studies using 3D echocardiography may provide more detailed evaluation of global RA function.

## Conclusions

RALS and RALSR measured by 2D-STE were useful for noninvasive evaluation of RA dysfunction and the severity of RHF in patients with PAH.
